# Handheld Echocardiography Measurements Concordance and Findings Agreement: An Exploratory Study

**DOI:** 10.3390/diagnostics13050853

**Published:** 2023-02-23

**Authors:** Mariam Haji-Hassan, Bogdan Duțu, Sorana D. Bolboacă

**Affiliations:** 1 Department of Medical Informatics and Biostatistics, “Iuliu Hațieganu” University of Medicine and Pharmacy Cluj-Napoca, Louis Pasteur Str., No. 6, 400349 Cluj-Napoca, Romania; 2 Cardiology Section, Clinical Rehabilitation Hospital, Viilor Str., No. 46-50, 400437 Cluj-Napoca, Romania

**Keywords:** handheld ultrasound (HH), heart imaging, point-of-care ultrasound, echocardiography

## Abstract

The professional association has already developed guidelines on the appropriate use of handheld ultrasound devices, especially in an emergency setting. Handheld ultrasound devices are seen as the ‘stethoscope of the future’ to assist in physical examination. Our exploratory study evaluated whether the measurements of cardiovascular structures and the agreement in identifying aortic, mitral, and tricuspid valve pathology made by a resident with a handheld device (HH, Kosmos Torso-One) reach the results reported by an experienced examiner who used a high-end device (STD). Patients referred for cardiology examination in a single center from June to August 2022 were eligible for the study. Patients who agreed to participate underwent two heart ultrasound examinations scanned by the same two operators. A cardiology resident performed the first examination with a HH ultrasound device, and an experienced examiner performed the second examination with an STD device. Forty-three consecutive patients were eligible, and forty-two were included in the study. One obese patient was excluded because none of the examiners succeeded in performing the heart examination. The measurements obtained with HH were generally higher than those obtained with STD, with the highest mean difference of 0.4 mm, but without significant differences (all 95% confidence intervals of the differences contain the value of 0). For valvular disease, the lowest agreement was observed for mitral valve regurgitation (26/42, with a Kappa concordance coefficient of 0.5321), which was missed in almost half of the patients with mild regurgitation and underestimated in half of the patients with moderate mitral regurgitation. The measurements performed by the resident with the handheld Kosmos Torso-One device showed high concordance with those conducted by the experienced examiner with a larger high-end ultrasound device. The learning curve of the resident could explain the limited performance in identifying valvular pathologies between examiners.

## 1. Introduction

Transthoracic echocardiography is the most common cardiac imaging modality [1], a central tool in diagnosing and managing heart disease. According to the EACVI (European Association of Cardiovascular Imaging) consensus, it has contributed to halving the frequency of major diagnostic errors over the last two decades [2].

The first handheld ultrasound device, Acuson P10 (Siemens), was launched in 2007 and commercialized as a diagnostic and screening tool for physicians and nurses. The following years have seen a decrease in both cost and dimensions of ultrasound devices. At the same time, the performance in image quality, available imaging modes, and measurement accuracy has continued to increase steadily [3].

Increased accessibility also makes handheld ultrasound devices attractive for teaching purposes. A study performed on Johns Hopkins Medical Center medical residents found that at least 30 scans are required to develop basic skills in heart ultrasound [4]. Reliable interpretation of ultrasound images and development of hand–eye coordination require repeated exposure. The reduced cost of handheld ultrasound devices, when compared to high end-devices (ranging between $50,000 and $100,000), means more people (residents and healthcare students) can benefit from increased exposure and practice time [5].

Several handheld ultrasound devices with specific features are available for performing echocardiography (Table 1).

The handheld devices presented in Table 1 have no continuous wave Doppler. Spectral Doppler is not featured on most handheld devices, as it causes them to heat up more rapidly [11]. Continuous wave Doppler is essential for diagnosing and grading valve stenosis. In addition, pulsed wave Doppler is needed in echocardiography, allowing quantitative assessment of left ventricular filling pressures (diastolic function). 

The Kosmos Torso-One (Echonous Inc.) is, as of September 2022, the only HH device that presents both pulsed wave and continuous wave capabilities [12]. Priced at $9300, Kosmos Torso-One has cardiac, abdominal, and lung ultrasound applications. Lexsa, priced at around $6000, is designed for vascular, musculoskeletal, and soft tissue ultrasound. 

Problem-oriented focus cardiac ultrasound examination (FoCUS) is recommended by international professional associations, especially in emergency settings (European Association of Cardiovascular Imaging [13], American College of Emergency Physicians [14], American Society of Echocardiography, and American College of Emergency Physicians [15]). Heart failure, shock, cardiac tamponade, cardiac arrest, respiratory distress, chest trauma, acute chest pain, and evaluation of valvular pathologies are the most relevant clinical presentations for FoCUS [16]. Guidelines on the appropriate use of handheld ultrasound devices are already being implemented (e.g., the Policy Statement of the American College of Emergency Physicians [17] or the European Society of Radiology [18]).

Handheld devices are sometimes seen as the ‘stethoscope of the future’ in the context of the decline of physical examination skills in the new entry physicians [19] and are recommended to be used as an instrument during physical examination [20]. The application-based and the knob-free platforms offer the advantage of accessible learning and quick adoption to all potential users. 

Evaluating handheld ultrasound devices in clinical practice is mandatory as an intermediate step towards current practice. The number of scientific publications reporting such devices’ performances in cardiovascular system evaluation is limited (see Table 2). The handheld devices were evaluated compared to a high-end ultrasound device used by examiners with similar experiences. The performances were higher on measurements than on diagnostic, and all studies showed deviations from the limits of agreements in Bland–Altman plots [21,22,23,24,25] (see also Table 2).

Handheld ultrasound devices have advantages and disadvantages and offer cardiology residents less intimidating and complex settings during the examination, negligible boot time, portability, and, sometimes, artificial intelligence guidance in measurements and identification of pathological findings [3]. However, how reliable are the measurements of heart structures performed by a cardiology resident compared to an experienced examiner? The primary objective of our exploratory study was to evaluate whether the measurements of cardiovascular structures made by a cardiology resident with a handheld device (HH, Kosmos Torso-One) reach the results reported by an experienced examiner who used a high-end device (STD). The secondary objective was to evaluate the agreement between a cardiology resident and an experienced cardiologist in identifying aortic, mitral, and tricuspid valve pathologies.

## 2. Materials and Methods

### 2.1. Study Design and Patients

We conducted an exploratory single-center prospective observational study in June–August 2022. We included consecutive adult patients admitted to the Cardiology ward, regardless of the presenting diagnoses, body mass index (BMI), or other factors. All patients were older than 18 years old and hemodynamically stable. The only exclusion criterion was the patient’s refusal to participate in the study. All patients signed an informed consent document.

### 2.2. Echocardiography Measurements

Patients underwent two heart ultrasound examinations and were scanned by the same two operators after the physical examination. The first examination (HH) was performed on the day of the presentation by a fifth-year cardiology resident using a Kosmos Torso-One (Echonous, Inc., Redmond, WA, USA) device with a phased array probe (three modes: heart, lung, and abdomen). The cardiology resident had previously received standard echocardiography training (a six-month cardiology imaging rotation). The second examination (STD) was performed the following day by an experienced cardiologist (>10 years of experience in echocardiography) using a high-end device with a phased-array cardiac probe (Philips 3300 CV, Philips Ultrasound Inc., Bothell, WA, USA) in the presence of the cardiology resident. The resident participated in the STD examination for training purposes and had no input into the STD report. Observation of the experienced examiner is valuable in the learning curve process, and the possibility to ask questions allows case-based training.

Regardless of the ultrasound device, we performed the ultrasound using the parasternal long and short axis views, apical 2-, 4-, and 5-chamber views, and subcostal views. In all cases, patients were positioned in the left lateral decubitus position. Images were optimized to provide optimal endocardial definition. Complete echocardiographic examination was performed in all patients, including bidimensional, color Doppler, pulsed wave Doppler, and continuous wave Doppler. Each examiner collected the measurements (diameter of the ascending aorta, end-diastolic diameter of the left ventricle, end-diastolic tickness of the interventricular septum, end-diastolic diameter of the left atrium, end-diastolic diameter of the right ventricle, left ventricular ejection fraction—LVEF), along with the information regarding the presence and severity of the valvular disease. Demographic data (e.g., age and sex) and referral diagnosis were also recorded for each examined patient.

**Figure 1:**
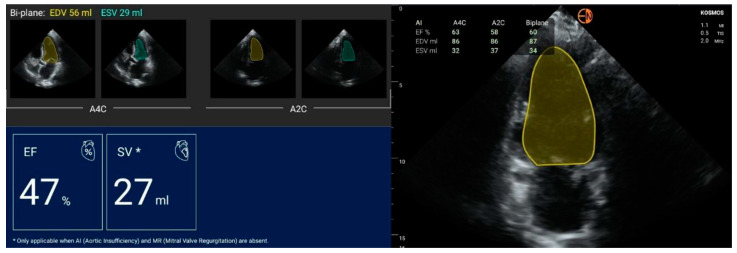
Automated calculation of ejection fraction (EF), end-systolic volume (ESV), end-diastolic volume (EDV), and stroke volume (SV) in a 57-year-old woman presenting with coronary artery disease.

The Kosmos Torso-One ultrasound device automatically calculates end-systolic volume, end-diastolic volume, and stroke volume (defined as the volume of blood pumped out of the left ventricle of the heart during each systolic cardiac contraction) in apical four-chamber and apical two-chamber view and provides an average between the two evaluations (Figure 1). We used this function for automatic contour detection and the determination of left ventricular parameters, including ejection fraction. However, the device allows users to manually trace the endocardial borders of the ventricles if they want to correct the automated measurements.

### 2.3. Statistical Methods

Variables were reported as mean (standard deviation) for age and number (%) for sex and referral diagnosis. The echocardiographic measurements were reported as mean (standard deviation), median [Q1 to Q3], and {min to max}, where Q1 is the 25th percentile and Q3 is the 75th percentile to allow an appropriate evaluation of the concordance between HH and STD. The agreement between two paired measurements (STD and HH) was reported as the difference between the gold standard measurement (STD) and HH measurement accompanied by the associated 95% confidence interval (95%CI). The difference is not statistically significant when 0 is in the 95% confidence interval. The visual representation of the agreement between measurements was reported using the Bland–Altman plot created with Real Statistics Using Excel (©Charles Zaiontz 2013–2022). 

The concordance of findings (qualitative data) was evaluated with the Kappa concordance coefficient using Linear Weighting (©Richard Lowry 2001–2022; http://vassarstats.net/kappa.html, accessed on 20 October 2022). A value of Kappa between 0.21 and 0.39 indicates a minimal agreement, between 0.40 and 0.59 indicates a weak agreement, between 0.60 and 0.79 indicates a moderate agreement, and between 0.80 and 0.90 indicates a strong agreement, while a value higher than 0.90 indicates an almost perfect agreement [26]. The values were expressed as point estimators and 95% confidence intervals (CI, expressed as lower to upper bounds). Student t-test for independent samples was used to compare the age of men and women. The comparison was made considering a two-tailed test, and a value less than 0.05 was considered statistically significant.

## 3. Results

Forty-three patients were consecutively included in the study with no refusal to participate, and forty-two were evaluated. One patient (male, 56 years old) with morbid obesity (BMI = 43 kg/m^2^) was excluded due to the impossibility of obtaining cardiovascular images either with the handheld or high-end transthoracic ultrasound probe. The excluded patient had been admitted for atrial fibrillation ablation and received the indication for transesophageal echocardiography, which was successfully performed.

### 3.1. Profile of the Patients

Patient ages ranged from 54 to 82 years old (average 65.4 (8.2) years). Most patients were men (25/42, 57.1%) younger than women (men vs. women—64.2 (8.3) years vs. 67.1 (8) years; Student t-test for independent sample: *p*-value = 0.2726). The diagnoses at the presentation were as follows: coronary artery disease—referred for invasive coronary imaging (14 patients, 33.3%), atrial fibrillation (7 patients, 16.7%), atrial flutter (2 patients, 4.8%), paroxysmal supraventricular tachycardia (1 patient, 2.4%) undergoing ablation, lower limb arteriopathy for peripheral angioplasty (6 patients, 14.3%), type 2 s-degree (4 patients, 9.5%) or third-degree (4 patients, 9.5%) heart block presenting for permanent heart stimulator implantation, dilated cardiomyopathy (4 patients, 9.5%), hypertrophic cardiomyopathy with signs and symptoms of decompensated heart failure (2 patients, 4.8%), and severe aortic stenosis (1 patient. 2.4%)

### 3.2. Measurements of Cardiovascular Structures

**Figure 2:**
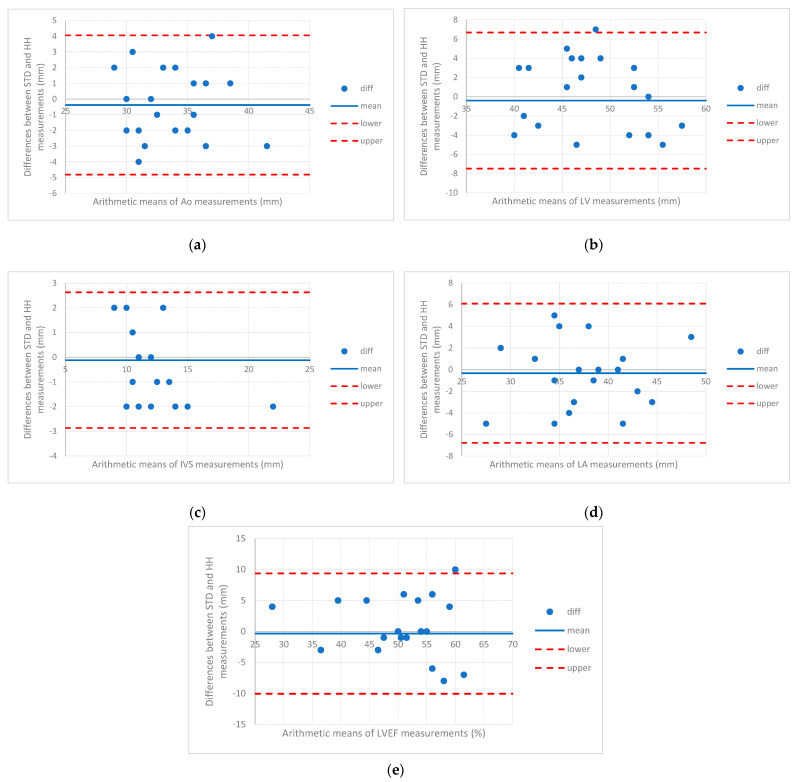
Bland–Altman plots for STD (conventional ultrasound)—HH (handheld ultrasound) interrater agreement analysis. Limits of the agreement are depicted as the dotted red line, the mean difference as the blue line, and pairs of differences between STD and HH as blue dots. (Ao = aorta, LV = left ventricle, IVS = InterVentricular Septum; LA = left atrium, LVEF = left ventricular ejection fraction). (**a**) diameter of the ascending aorta; (**b**) end-diastolic diameter of the left ventricle; (**c**) end-diastolic interventricular septum tickness; (**d**) end-diastolic diameter of left atrium; (**e**) left ventricular ejection fraction.

A good agreement between the HH and STD measurements was observed, with only two values that exceeded the upper bound of agreement (one measurement of LV—left ventricle and one of LVEF—left ventricular ejection fraction) (Table 3, Figure 2). The LV was 52 mm as measured with STD and 45 mm when measured with HH, with a difference of 7 mm, while the upper bound was 6.7 with a 95% confidence interval from 4.7 to 8.6. Like LV, the LVEF had higher values when measured with STD (65%) than HH (55%), with a difference of 10%, a value that did not exceed the upper bound of the upper value in the Bland–Altman plot (94, 95%CI from 6.7 to 12.1).

### 3.3. Valvular Stenosis and Regurgitation

The smallest agreement between STD and HH was observed for the pathology of the mitral valve (Table 4). 

**Figure 3:**
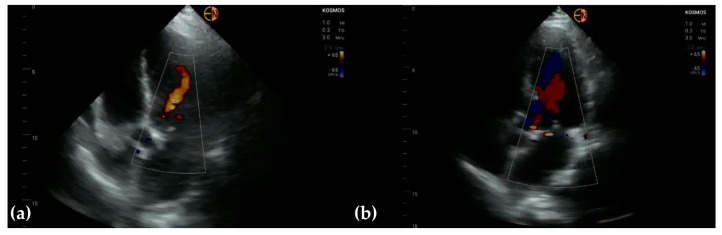
(**a**) Apical 5-chamber view in 63-year-old woman identifies aortic regurgitation, classified as mild on HH examination; STD confirmed moderate aortic regurgitation, based on vena contracta measurement and PHT; (**b**) apical 5-chamber view in 68-year-old woman identifies minimal aortic and mitral regurgitation (HH); STD classified both valvular regurgitations as moderate.

**Figure 4:**
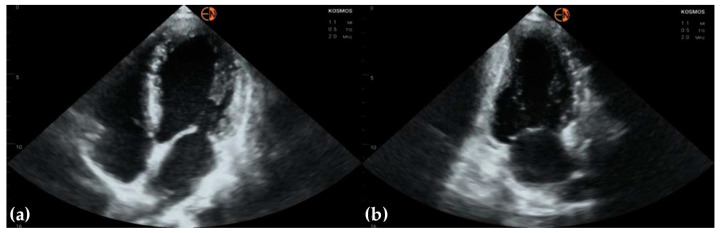
(**a**) A4C and (**b**) A2C view on a patient presenting with peripheral arteriopathy (angioplasty of left superficial femoral artery). The mitral valve was classified as having no regurgitation on handheld examination, while conventional ultrasound showed mild mitral regurgitation.

Half of the cases with mild mitral regurgitation were not identified on handheld ultrasound examination (8/16). Five subjects with moderate mitral insufficiency were also underestimated (5/10). Thirteen of fifteen patients with no mitral insufficiency were correctly identified. All cases of aortic stenosis were appropriately identified by handheld ultrasound, but only 3/10 patients with mild aortic regurgitation were correctly identified (7/10 were classified as normal) (Figure 3 and Figure 4). 

**Figure 5:**
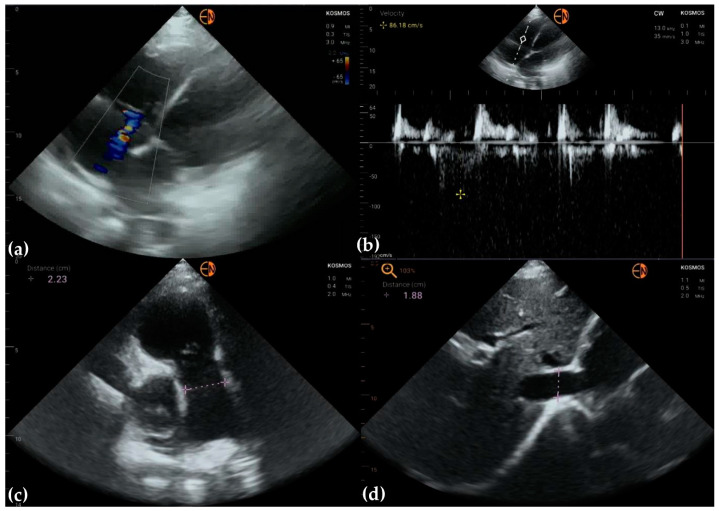
(**a**) Moderate tricuspid regurgitation is correctly identified on color-Doppler in a 72-year-old man presenting with decompensated heart failure; (**b**) continuous wave Doppler in the same patient produces faint Doppler envelope at the tricuspid valve, making pulmonary artery pressure estimation difficult; on STD, the pressure gradient between the right ventricle and right atrium was increased; (**c**) pulmonary artery and (**d**) inferior vena cava measurements.

The cardiology resident appropriately diagnosed, with HH ultrasound, 10 out of 19 patients with mild or moderate tricuspid valve insufficiency, underestimated the severity of insufficiency in 4 out of 7 patients with moderate tricuspid regurgitation, and missed tricuspid regurgitation in 5 out of 12 patients with mild regurgitation (Figure 5). Eighteen out of twenty tricuspid valves without regurgitation were correctly classified by the cardiology resident using the HH device.

### 3.4. Other Findings

**Figure 6:**
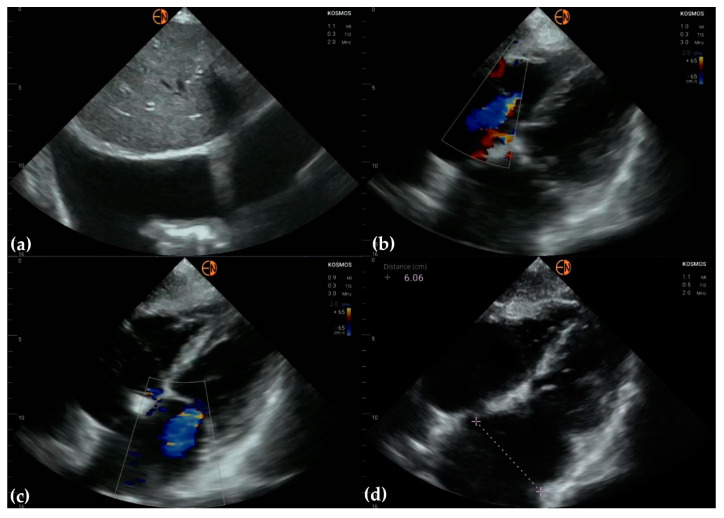
(**a**) Mild right pleural effusion in a patient presenting with signs and symptoms of decompensated heart failure; (**b**) moderate tricuspid regurgitation; (**c**) severe mitral regurgitation; (**d**) enlarged left atrium.

A pleural effusion was found in one patient. It was a mild right pleural effusion in a 78-year-old male who presented for dyspnea and orthopnea and congestive heart failure with severe left ventricular systolic dysfunction, severe mitral regurgitation, and moderate tricuspid regurgitation (Figure 6).

**Figure 7:**
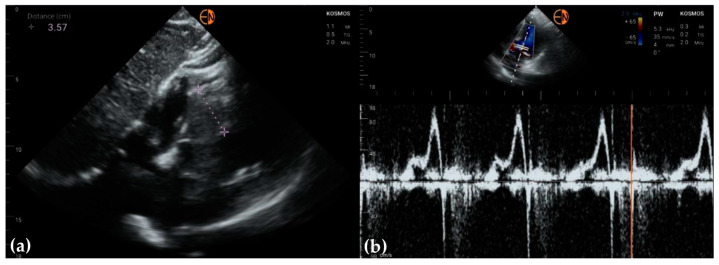
(**a**) Severe interventricular septum hypertrophy in a 58-year-old woman presenting for dyspnea caused by hypertrophic cardiomyopathy; (**b**) trans-mitral flow showing diastolic dysfunction in the same patient.

The examination with the handheld device missed no cases of significant left ventricular hypertrophy, dilatation, or impaired systolic function (Figure 7).

**Figure 8:**
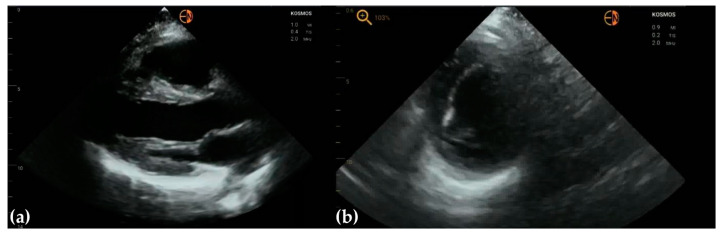
(**a**) Parasternal long axis view in a 57-year-old man presenting with aggravated chest angina shows an appropriate delineation of endocardial borders, allowing optimal measurement of heart structures and evaluation of LV motion; (**b**) parasternal short axis view shows obscuring of the LV antero-lateral wall in a 72-year-old man who was a smoker and presented with chest angina, probably because of lung reverberation effect; this was not noticed with the high-end device.

Figure 8a shows a patient where endocardial borders are clearly visualized. Figure 8b shows a patient in whom the antero-lateral wall of the left ventricle was blurred in the images obtained with the handheld device, which made analysis of the kinetic function of the left ventricle challenging. In such patients, hypokinesis of the affected segments was suspected but disproven by the high-end examination.

**Figure 9:**
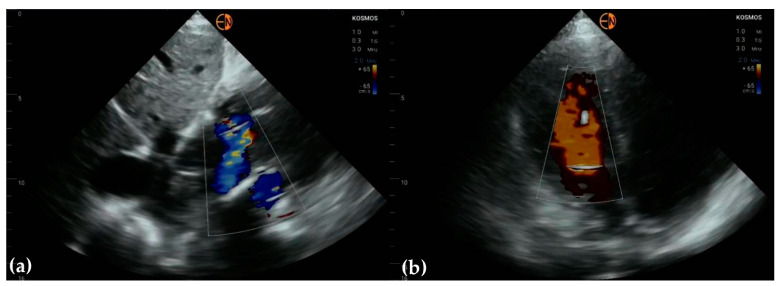
(**a**) Para-lead severe tricuspid regurgitation in a 67-year-old man with dual chamber stimulation leads; (**b**) visualization of stimulation lead in a 78-year-old woman who presented with high-degree atrio-ventricular block.

Intracardiac devices and significant para-lead regurgitations were clearly visualized in our study (Figure 9).

**Figure 10:**
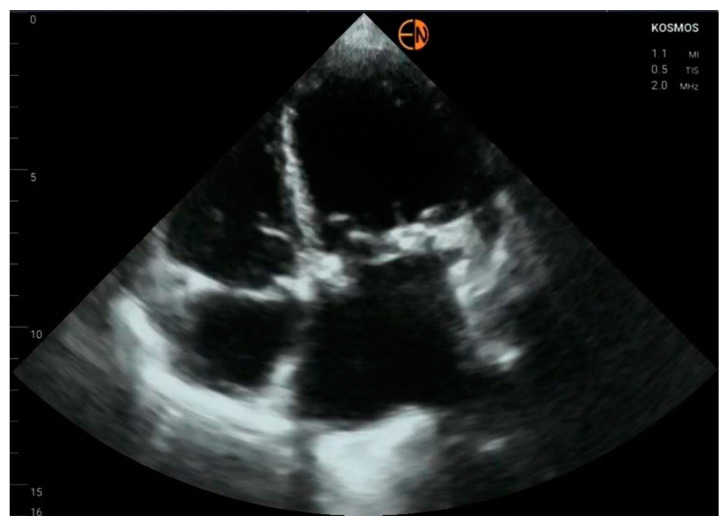
Mitral valve biological prosthesis in a 71-year-old man shows thickened valve cusps with reduced motion.

The Kosmos Torso-One ultrasound device did not allow quantitative measurements for evaluating mitral valve stenosis and mitral prosthesis function when the study was conducted. In a patient with a mitral biological prosthesis, thickened valve cusps and reduced motion suggested malfunction of the prosthetic valve but did not provide measurements for quantification of the dysfunction. Trans-mitral mean pressure and pressure half-time were performed with the high-end ultrasound device and were consistent with moderate stenosis of the mitral biological prosthetic valve (Figure 10).

**Figure 11:**
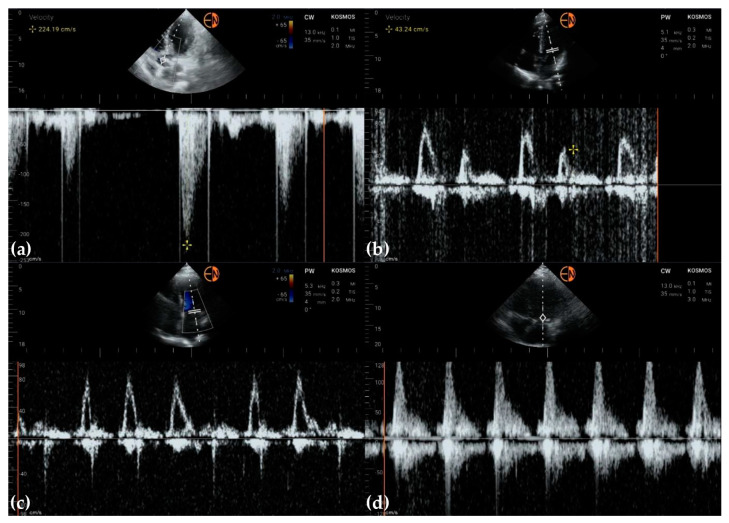
(**a**) Continuous wave Doppler shows post-extrasystolic aortic velocity; (**b**) pulsed wave Doppler of mitral flow in a patient with normal diastolic LV function; tissue Doppler imaging is not yet available on any handheld ultrasound, to differentiate from pseudo-normal diastolic function; (**c**) absence of A waves in a patient with long-standing atrial fibrillation; (**d**) trans-mitral flow with absent A wave in a man presenting with 2:1 atrial flutter.

Pulsed wave and continuous wave Doppler were used to quantify blood flow through valves (Figure 11).

## 4. Discussion

Our study shows an appropriate concordance of cardiovascular structure measurements between STD and HH devices, considering the difference between the examiners (cardiology resident vs. experienced cardiologist). The concordance between diagnoses was smaller than between measurements, showing weak to moderate agreements. The observed difference could be explained not only by device performance but also by the resident’s learning curve and the experienced echocardiographer’s expertise.

No studies were available in the scientific literature to report the performances of the Kosmos device used in our study (Table 2) when we wrote our study protocol. Le et al. compared four handheld ultrasound devices: Butterfly iQ+ by Butterfly Network Inc., Kosmos by EchoNous, Vscan Air by General Electric, and Lumify by Philips Healthcare. While all these devices have bidimensional and color Doppler modes, only Kosmos features spectral Doppler, making it the only device suitable for quantification of valvular stenoses, ventricular diastolic filling, and pulmonary artery pressure [10]. The Kosmos device is the only one that offers both pulsed wave and continuous wave Doppler (Figure 11).

Our results agree with other studies that compared pocket-size devices with conventional transthoracic examinations. Jenkins et al. [27] reported in a meta-analysis (33 studies and 6062 patients) that pooled sensitivities for reduced left ventricular ejection fraction, wall motion abnormalities, left ventricular dilatation, and left ventricular hypertrophy ranged between 85% and 89%, while pooled specificities were between 91% and 98%. Less experienced operators could detect cardiac abnormalities with reasonable sensitivity and specificity, but with a significant difference in diagnostic accuracy compared to experienced users [28]. As in our findings, Blume et al. [25] reported lower concordance regarding mitral, aortic, and tricuspid regurgitations but with higher differences between STD and VScan regarding the LVEF (6 points outside the tolerance of 5% difference).

Blurred endocardial borders represented a common issue in our study, more frequently encountered with handheld ultrasound, leading to differences in the measurement of heart chambers between handheld (HH) and high-end (STD) devices. Generally, the cardiovascular measurements had, in our study, higher HH values than STD for LVEF, Ao, and LV, but with identical measurements for IVS (Table 2). Despite these differences in the concordance of measurements, only in a few cases were the values smaller than the lower bound or higher than the upper bound (Figure 2), showing a good agreement considering that ultrasound examination is operator-dependent. An EACVI consensus on the use of handheld ultrasound devices in 2018 stated that linear measurements of most cardiac structures and vessels could be performed reliably, and the concordance with measurements from high-end systems is usually good if the examiners are appropriately trained. However, according to the same position statement, the lack of spectral Doppler prevents a comprehensive quantitative assessment of valve disease severity and systolic pulmonary artery pressure [28]. The above-presented shortcomings have been overcome with the appearance of handheld devices with spectral Doppler capabilities. In our study, the cardiology resident missed many cases of mild valvular regurgitation, while some moderate or severe regurgitation cases were underestimated. The underestimation could be explained by the difference in experience between examiners and the inferior quality of color Doppler on the handheld device compared to the conventional ultrasound device. Moreover, pulmonary artery pressure was sometimes underestimated due to the faint continuous Doppler envelope at the tricuspid valve. We also could not measure pulmonary artery acceleration time with the handheld ultrasound device, another limitation of the Echonous Kosmos Torso-One device.

One disadvantage of using a handheld ultrasound device is its incapacity to generate the same image quality as the larger device. Image HH quality was also more prone to artifacts than the high-end (STD) counterpart. Wilkinson and Saxhaug [3] raise concerns over possible mismanagement of patients because of poor ultrasound images on a small screen. They see portable ultrasound as most likely beneficial for training ultrasonographers and mention the increased use of such devices in medical universities. The first step in learning to use ultrasound is image acquisition, and training in hand–eye coordination requires hours of practice. However, the setting needed for image acquisition with a HH device is minimal and somehow standardized, making HH devices ready to use with less training compared to standard ultrasound devices.

According to the Echonous representatives, when they first started developing the handheld ultrasound device, they mainly focused on providing a learning tool for medical students [29]. This would most likely happen if medical universities offered access to these portable ultrasound devices, because their price is still prohibitive for most medical students. However, the handheld device must be carefully chosen, as poor image quality may negatively affect the ability of users to attain competency.

Currently, the main application of artificial intelligence in the Kosmos device is automated ejection fraction calculation. Papadopoulou et al. [30] compared the ejection fractions calculated by the Kosmos device with the ejection fractions obtained by the manual biplane Simpson’s method on a larger ultrasound device, showing fast and reliable results. The future of handheld ultrasound devices holds important developments, adding machine learning-driven software that automates the analyses of echocardiographic imagery and measurements. If this happens, inter-operator variability regarding image interpretation could be reduced. Furthermore, the HH can become a reliable assistant in diagnosis to reduce false positive and false negative results.

Our study has its limitations, the most important ones being the applied design, namely, exploratory study, and thus the limited number of included patients. Secondly, in every patient, the examination with the handheld ultrasound device was performed by a cardiology resident, while the sonographist performing the echocardiography with the high-end device had more than ten years of experience. The applied design of the study limited the intra-observed reliability analysis on different devices (agreement and concordance analysis of the HH and STD evaluation conducted by the same examiner). Furthermore, the results reported on inter-observer reliability are biased by the experience of the examiners, so the difference in experience may account for some of the differences between evaluations. Cardiac ultrasound examination is operator-dependent, so differences even between two experienced examiners when using the same device are common [21,22,23,24]. Limitations of the device settings impose a focus of the resident on the physical examination; this could lead to a higher concordance of measurements compared to measurements taken by the resident with an STD. Second, as an exploratory study design, we evaluated a limited number of patients. In this regard, our results support the usefulness of a more extensive study to include patients with appropriate variability in all confounder variables (e.g., body mass index, cardiologic pathology, etc.) to evaluate clinical performances properly. Furthermore, we did not address the technical characteristics of the probe, such as battery life, overheating, compatibility with other devices, and image archiving options. All these technical characteristics are essential when the number of evaluated patients increases to ensure a timely examination in real-life healthcare settings and need to be assessed in further clinical studies.

## 5. Conclusions

The measurements of cardiovascular structures taken by a cardiology resident with a Kosmos Torso-One device showed high concordances with the larger high-end ultrasound device used by an experienced examiner. The learning curve could explain the limited performance in identifying valvular pathologies by the resident with the Kosmos Torso-One device. Incorporating algorithms in assisting measurements and identifying pathological findings could make echocardiographic examinations less operator-dependent and assist even less-experienced examiners in daily practice to ensure timely and accurate patient triage and/or diagnosis.

## Figures and Tables

**Table 1:** Features of some handheld ultrasound devices.

HH Device [Ref]	Price	Features
Vscan Extend with Dual Probe (GE’s Vscan) [6] *	around €5100 (plus taxes)	B-mode, M-mode, and color flow imaging at a frequency between 1.7 and 3.8 MHz, with a scan time of 60 min and a recharge time of 75 min
Vscan Air (GE’s Vscan) [6] *	€4.250 (plus taxes)	similar to Vscan Extend with Dual Probe but has a wireless probe
Clarius [7] *	around €3395 require a €595 annual membership fee	bidimensional and color Doppler imaging capabilities
Philips’ Lumify [8] *	around €5800 per probe + cost for the tablet	three probes: linear features pulsed wave Doppler, convex, and phased array—used for cardiac imaging, does not provide continuous wave Doppler
Butterfly iQ [9] **	around $2000 plus an annual membership fee	sacrificed image quality, especially in the examination of the heart [10]

HH = handheld device; * use classic piezoelectric crystal technology; ** silicone-chip technology (called ultrasound-on-a-chip).

**Table 2:** Handheld ultrasound evaluation of cardiac morphology and function.

Who?	HH Device	TTE (Gold Standard)	n	Examiners	Reported Results
Prinz et al. [21], 2012	Vscan (GE Vingmed Ultrasound, Horten, Norway)	Vivid 7system (GE Vingmed Ultrasound	320 inpatients	2	Concordance: low for “mild” regurgitant valvular lesions; Agreement: positive and negative deviation on Bland–Altman agreement plots for LVEF
Cullen et al. [22], 2014	Vscan (GE Healthcare)	iE33 (Phillips) or Vivid E9 (GE Healthcare)	190 outpatients	6 *	Agreement: from 0.51 (0.28 to 0.74) for increased LV wall thickness (present vs. absent) to 0.91 (0.86 to 0.95) for aortic diameter (mm)
Cullen et al. [23], 2017	Vscan (GE Healthcare, Waukesha, WI)	Vivid E9 (GE Healthcare, Waukesha, WI) or iE33 (Philips Healthcare, Andover, MA)	82 patients with acute myocardial infarction	2	Concordance: 0.75 (0.66 to 0.82) for LEFV; 0.69 (0.58 to 0.77) for WMSI; Agreement: 0.55 to 0.79 for mitral regurgitation and 0.48 to 0.78 for tricuspid regurgitation
Acheampong et al. [24], 2020	Lumify	Philips EPIQ7	60 pediatric outpatients	unclear	Agreements were smaller for children ≤5 years (e.g., 0.59 (0.31 to 0.88), n = 20 for fractional shortening compared to 0.82 (0.73 to 0.90) for all patients (n = 55))
Blume et al. [25], 2022	Vscan (GE Vingmed Ultrasound AS, Horten, Norway)	Philips iE33 (Philips Ultrasound Inc., Bothell, WA, USA)	108 patients	2	Concordance: from 0.25 (0.17 to 0.32) for pulmonary regurgitation (none/mild/moderate/severe) to 0.86 (0.80 to 0.90) for LEFV

HH = handheld echocardiography; TTE = Transthoracic echocardiography; n = number of evaluated patients; LEFV = left ventricular ejection fraction; 95%CI = 95%confidence interval (lower bound to upper bound); WMSI = wall motion score index; * one HH examination per patient; LV = left ventricle.

**Table 3:** Agreement between HH and STD echocardiography measurements.

Item	Statistics	STD	HH	Diff.	Agreement [95%CI]
Ao (mm)	mean (stdev)	34 (3.8)	34.4 (4)	−0.38 (2.26)	−0.4 [−4.8 to 4.1]
	median [Q1 to Q3]	35 [32 to 36]	35 [32.3 to 36]	−0.5 [−2 to 1]	
	{min to max}	{24 to 41}	{25 to 43}	{−4 to 5}	
LV (mm)	mean (stdev)	48.1 (5.4)	48.5 (5.7)	−0.4 (3.61)	−0.4 [−7.5 to 6.7]
	median [Q1 to Q3]	48 [44.3 to 52.8]	47 [44.3 to 53.8]	−1 [−3 to 2.75]	
	{min to max}	{35 to 56}	{36 to 59}	{−6 to 7}	
IVS (mm)	mean (stdev)	12 (2.2)	12.1 (2.7)	−0.12 (1.4)	−0.1 [−2.9 to 2.6]
	median [Q1 to Q3]	12 [10.3 to 13]	12 [11 to 13]	0 [−1 to 1]	
	{min to max}	{9 to 21}	{8 to 23}	{−2 to 3}	
LA (mm)	mean (stdev)	39.2 (5.9)	39.5 (5.8)	−0.33 (3.28)	−0.3 [−6.8 to 6.1]
	median [Q1 to Q3]	39 [35 to 42]	39.5 [36.3 to 44]	0 [−3 to 2.75]	
	{min to max}	{25 to 50}	{28 to 51}	{−7 to 5}	
FEVS (%)	mean (stdev)	51.4 (9.1)	51.7 (9.8)	−0.33 (4.96)	−0.3 [−10.1 to 9.4]
	median [Q1 to Q3]	53.5 [46 to 57]	52.5 [48 to 58.5]	−1 [−3 to 4]	
	{min to max}	{30 to 67}	{26 to 68}	{−11 to 10}	

STD = conventional ultrasound; HH = handheld ultrasound; Ao = aorta, LV = left ventricle, IVS = InterVentricular Septum; LA = left atrium, LVEF = left ventricular ejection fraction; stdev = standard deviation; Q1 = the 25th percentile; Q3 = the 75th percentile; min = minimum value; max=maximum value; Diff = the difference between STD and HH; 95%CI is the 95% confidence interval for the agreements expressed as [lower bound to upper bound].

**Table 4:** Agreement between STD and HH on qualitative data.

	STD	HH	Full Agreement n (% [95%CI]) Kappa * [95%CI]
Aortic valve			33 (78.6 [62.8 to 89.2]) 0.6557 [0.3862 to 0.9252]
Mild aortic regurgitation	10	5	
Moderate aortic regurgitation	1	0	
Mild aortic stenosis	1	1	
Severe aortic stenosis	1	1	
Normal	29	35	
Mitral valve			26 (61.9 [45.7 to 76.0]) 0.5321 [0.3451 to 0.7191]
Mild mitral regurgitation	16	14	
Moderate mitral regurgitation	10	7	
Severe mitral regurgitation	1	1	
Normal	15	21	
Tricuspid valve			29 (69.0 [52.8 to 81.9]) 0.6072 [0.4183 to 0.7961]
Mild tricuspid regurgitation	12	11	
Moderate tricuspid regurgitation	7	5	
Severe tricuspid regurgitation	3	1	
Normal	20	25	

Data are reported as numbers; * Kappa with Linear Weighting.

## Data Availability

The data that support our findings are in the manuscript. The raw data are not publicly available due to privacy restrictions but could be obtained from the first author based on reasonable request.
